# Molecular data reallocates *Sorosphaerula radicalis* (Plasmodiophorida, Phytomyxea, Rhizaria) to the genus Hillenburgia

**DOI:** 10.1111/jeu.12924

**Published:** 2022-06-13

**Authors:** Michaela Hittorf, Martin Kirchmair, Andrea Garvetto, Sigrid Neuhauser

**Affiliations:** ^1^ Institute of Microbiology University of Innsbruck Innsbruck Austria

**Keywords:** 18S rRNA phylogeny, *Hillenburgia*, Phytomyxea, plant pathogen, plasmodiophorids, *Sorosphaerula radicalis*

## Abstract

This study reports the first record of *Sorosphaerula radicalis* (Phytomyxea, Rhizaria) in continental Europe (Tirol, Austria) and provides first molecular data for this species. An 18S rRNA phylogeny placed *S. radicalis* into the Plasmodiophorida, although distant from other members of the genus *Sorosphaerula* and close to the parasite of water cress *Hillenburgia nasturtii*. To resolve this polyphyly, we compare morphological data and life cycles of *Sorosphaerula veronicae* (the type species of the genus *Sorosphaerula*), *Hillenburgia nasturtii,* and *Sorosphaerula radicalis*. We conclude that *Sorosphaerula radicalis* belongs to the recently established genus *Hillenburgia*.

Phytomyxea (SAR, Rhizaria) are a group of obligate biotrophic parasites (Bass et al., 2009). Currently, the Plasmodiophorida, the Phagomyxida, and the newly discovered Marinomyxa/Tagiri5 clade belong to this group (Bass et al., 2009; Cavalier‐Smith & Chao, 2003; Kolátková et al., 2021). Some species like *Plasmodiophora brassicae* or *Spongospora subterranea* are well studied because they infect economically important plants (Neuhauser et al., 2010). *Plasmodiophora brassicae,* the cause of clubroot disease, infects Brassicaceae, including economically important varieties in the species *Brassica oleracea*, *B. napus* and *B. rapa* (Hwang et al., 2012) and causes worldwide crop losses of up to 10% (Dixon, 2009). *Spongospora subterranea* leads to significant losses in potato production worldwide (Balendres et al., 2016) causing powdery scab of potatoes but also as vector of the potato mop‐top virus (PMTV) (Beuch et al., 2015). Virus transmission is a common feature of phytomyxids and these viruses are responsible for crop loss in grain and sugar beet production (Abe & Tamada, 1986; Kanyuka et al., 2003; Lubicz et al., 2007). *Hillenburgia nasturtii* (originally classified as *S. subterranea* f.sp. *nasturtii* by Tomlinson in, 1958) is the causal agent of crook root in water cress and transmits the watercress yellow spot virus (WYSV) (Tomlinson, 1958; Walsh et al., 1989). However, phytomyxea infecting plants in the natural environment are often overlooked and reports of them are rare, although many of them appear to be quite widespread and abundant (Bulman & Braselton, 2014; Neuhauser et al., 2011). Many of the species described during the 19th and 20th century have been found only occasionally and by chance, resulting in a gap of knowledge about those species. An example is *Pseudoligniera verrucosa,* for which recent molecular data have been produced, allowing its assignment to a novel genus distant from its original placement within the genus *Ligniera* (Hittorf et al., 2020).

Another elusive phytomyxean species is *Sorosphaerula radicalis,* an obligate intracellular parasite of Poacea originally described by Cook and Schwartz (1929). It is one of the three accepted species belonging to the genus *Sorosphaerula* (Neuhauser & Kirchmair, 2011): *S. veronicae* (Schröter, 1877), *S. radicalis* (Cook & Schwartz, 1929) and *S. viticola* (Neuhauser et al., 2009). The type species *S. veronicae* is well characterized by morphological and cytological studies (Blomfield & Schwartz, 1910; Miller, 1958), studies on the ultrastructure (Braselton et al., 1975; Braselton & Miller, 1973; Braselton & Miller, 1975) and through 18S rRNA phylogenies (Bulman et al., 2001; Hittorf et al., 2020; Neuhauser et al., 2014). *S. viticola* is characterized by ultrastructural and morphological studies (Kirchmair et al., 2005; Neuhauser et al., 2009) and 18S rRNA phylogenies (Neuhauser et al., 2014). *Sorosphaerula ulei* (Schröter, 1896) was originally as belonging to this genus but it is conspecific with *Sorodiscus callitrichis* (Liro, 1935) and therefore excluded from the genus. The fifth described species of *Sorosphaerula*, *S. urticae* was found only once and its description is very superficial and lacking any figures (Naumov, 1954). It is therefore regarded as a doubtful species. *Sorosphaerula radicalis* has been well documented by Cook and Schwartz (1929) with a comprehensive description complete with micrographs; however, no contemporary images or DNA sequences of this species are available. Here, we provide the first 18S rRNA sequence of *S. radicalis*, revise its taxonomic position, and provide an extended description of its life cycle.

## MATERIAL AND METHODS

### Sampling

Plant samples (*Veronica* sp. and *Poa* sp.) were collected from a sandy bank of the river Melach (Kühtai, Tirol, Austria) on the first of June 2021 as part of a sampling campaign aiming to find different phytomyxean species. Roots of the samples were rinsed with tap water and screened for infections with phytomyxea using a Nikon Optiphot 2 light microscope.

### Microscopy

Roots were fixed in 4% Histofix (phosphate‐buffered formaldehyde solution; Carl Roth) for one hour. Samples were dehydrated in an ascending ethanol series (50% EtOH, 2 × 70% EtOH, 100% EtOH) before being incubated in Hoechst 33342 for 10 min, mounted in Vectashield (H‐1000, Vector Laboratories), and analyzed using epifluorescence microscopy. Alternatively, propidium iodide was used for DNA staining on Histofix‐preserved samples by mounting them directly with ROTI®Mount FluorCare PI (Carl Roth). Samples were analyzed using a Nikon Eclipse Ti2‐E microscope equipped with an Andor Zyla 5.5sCMOS monochrome camera and Nikon CFI Plan‐Fluor 40×/0.75 NA and 60×/0.85 NA objectives using the excitation wavelengths 365 nm (for Hoechst 33342) and 535 nm (for propidium iodide). The NIS Elements software (Nikon) was used for imaging analysis (overlaying images from the DIC channel with images from the fluorescent channels for Hoechst or PI). The image plates presented in this paper were assembled using Inkscape 0.92.4.

All measurements are given in the form (minimum) mean ± standard deviation (maximum).

### DNA extraction

Infected roots were homogenized using a FastPrep‐24™ 5G (Biomedicals) at 6.0 m/s for 40 s. DNA was extracted using the DNeasy Plant Mini Kit according to the manufacturers’ instructions (Quiagen). DNA extracts were stored at −20°C until further use. The primers used were s4f (5′‐GGCAGCAGGYGYGHAAATIRYCCA‐3′) and C9rPhyt (5′‐GGAATTCCTCGTTGGTGCG‐3′) (Hittorf et al., 2020). Each PCR mix (29.3 μl) included final concentrations of 0.2 mM dNTP mix (Fermentas), 1 μM of each primer, 3 mM MgCl2 (Promega), 1× GoTaq flexi buffer (Promega), 2 mg/ml BSA (Sigma‐Aldrich) and 0.25 U GoTaq DNA polymerase (Promega). 2 μl DNA extract was used for each reaction and a negative control was included. PCR conditions were 95°C for 3 min, followed by 33 cycles of 95°C for 30 s, 65°C for 30 s and 72°C for 90 s. This was followed by a 10‐min final elongation step at 72°C. PCR products were visualized on a 1% agarose gel (Biozym Scientific GmbH) stained with 0.1 μl of SYBR Safe (Thermo Fisher Scientific) per 30 ml. PCR products were purified using a PEG purification (Neuhauser et al., 2014) and sent to Eurofins Genomics Sequencing GmbH for sequencing.

### Phylogenetic analysis

Sequences were aligned to the alignment of phytomyxean 18S rRNA sequences from Hittorf et al. (2020) using MAFFT v7.308 (Katoh & Standley, 2013) as implemented in Geneious v.9.0.5 (Kearse et al., 2012) with default parameters. The dataset was analyzed using IQ‐Tree 1.6.12 (Nguyen et al., 2015) using the TIM2 + F + R4 model identified as the best substitution model using ModelFinder (Kalyaanamoorthy et al., 2017). Ultrafast bootstrap with 1000 replicates (Hoang et al., 2018) was used to compute branch support in phylogenetic reconstructions using IQ‐Tree. The trees were visualized and annotated using the online tool Interactive Tree Of Life (iTOL) version 6.3 (Letunic and Bork, 2021) (https://itol.embl.de/) and annotations were added in Inkscape 0.92.4. The novel sequences were deposited in NCBI GenBank under the accession numbers OM914409 and OM914410.

## RESULTS

### Microscopy

**FIGURE 1:**
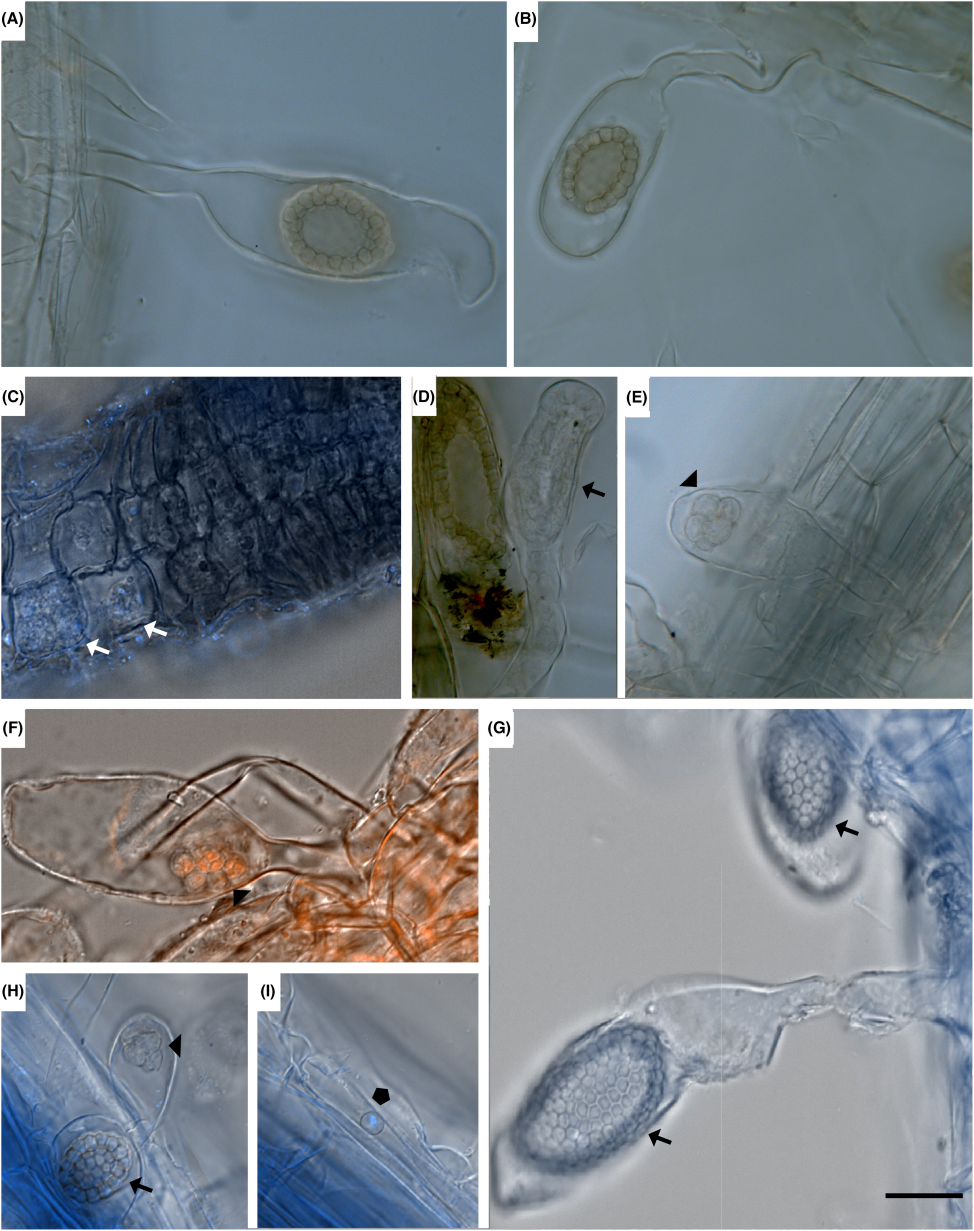
Life cycle stages of *Hillenburgia radicalis*. (A, B) Typical hollow oval brown/ochre colored sporosorus in hypertrophied root hair (unstained sample). (C) Multinucleate plasmodia (white arrows) in the root elongation zone. Nuclei are stained with Hoechst (blue). (D) Developing sporosorus. (E) Zoosporangium (arrowhead) unstained. (F) Zoosporangium (arrowhead), nuclei are stained with propidium iodide (orange). (G) Enlarged root hairs with thick‐walled hexagonal shaped resting spores forming sporosori (arrows). (H) Different life cycle stages of *H. radicalis*: sporosorus (arrow), zoosporangium (arrowhead). (I) Putative encysted spore ready for infection (pentagon). (A), (B), (D), (E): Light microscopy, sample unstained in water; (F): DIC picture, propidium iodide staining (orange). (C), (G–I): Differential interface contrast (DIC) pictures; nuclei are stained with Hoechst. Scale bar = 20 μm

*Sorosphaerula radicalis* was found in root hairs and in the root epidermis of *Poa* sp. The resting spores are thick‐walled, (3.5) 4.1 μm ± 0.3 (4.9) × (2.5) 3.3 μm ± 0.3 (3.9) in diameter (*n* = 60) and aggregated into hollow sporosori (Figure 1A, B, D, G, H). The sporosori are oval to round varying in size and diameter (15) 25.7 μm ± 10.2 (64.2) × (13) 18.1 μm ± 3.7 (27.5) (*n* = 21). One layer of resting spores encloses the hollow center in the middle of the sporosorus. Sporosori are found in root hairs and very rarely in the root epidermis. The sporosori can be easily spotted by their distinctive brownish/ochre color (Figure 1A, B). Thin‐walled zoosporangia were observed in enlarged root hairs (Figure 1E, F, H) and plasmodia were found in epidermal cells near the root tip (Figure 1C). Encysted spores were found on root hairs and on epidermal cells in the root elongation zone and the root tip (Figure 1I, polygon). *Sorosphaerula radicalis* causes hypertrophy in infected root hairs but no additional symptoms were observed on above and below ground plant parts. The slight reddening of the stem and leaf bases mentioned by Cook and Schwartz 1929 could not be observed.

### Molecular phylogenetic analysis

**FIGURE 2:**
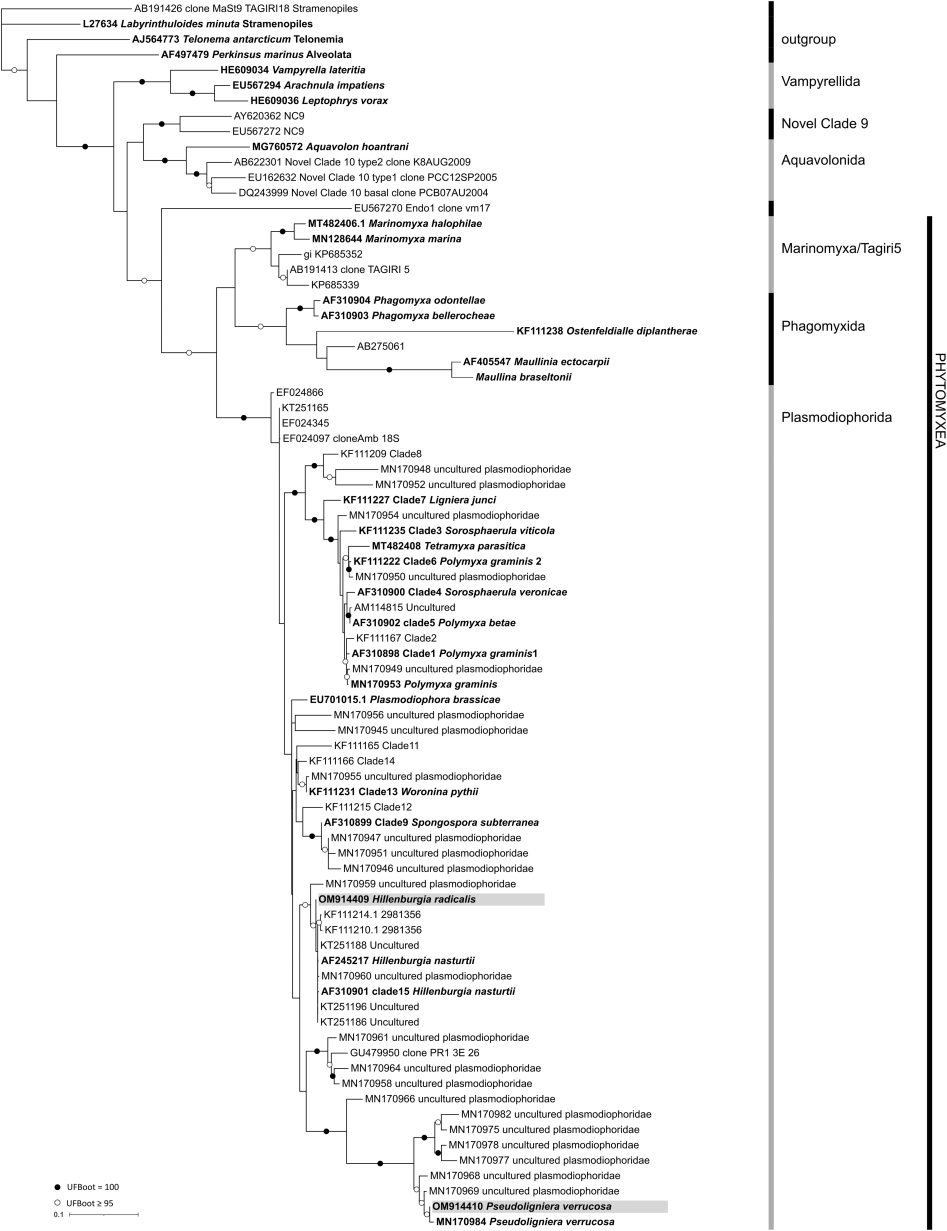
18S rRNA phylogenetic tree (maximum likelihood) of the class Phytomyxea including the new sequence of *Hillenburgia radicalis*. 80 sequences, 861 positions. Sequences from specimen are highlighted in bold letters; new sequences are highlighted with a gray background. Ultrafast Bootstrap support is shown as white dots if 0.95 or higher and as black dots if 1.00

In the phylogenetic analyses, all main clades of the Phytomyxea were resolved and well supported (Figure 2). The closely related Vampyrellida, Novel Clade 9, and the Aquavolonida had a maximum ultrafast bootstrap support (UFBoot = 100), while the Phytomyxea were supported with an UFBoot of 98. The three main lineages within the Phytomyxea were robustly supported: the Plasmodiophorida (UFBoot = 100) and the Phagomyxida and the Tagiri5/Marinomyxa (UFBoot = 99). The sequence from a novel *Pseudoligniera verrucosa* isolate (OM14410) clustered robustly with sequences from other *P. verrucosa* sequences (identity of 99.5% with MN170984). *Sorosphaerula veronicae* and *S. viticola* cluster together with *Polymyxa graminis*, *Polymyxa betae*, *Tetramyxa parasitica,* and *Ligniera junci*. The sequence of the isolate of *S. radicalis* clusters together with *Hillenburgia nasturtii* and seven environmental sequences (KF111214.1, KF111210.1, KT251188, MN170960, KT251196, KT251186, MN170959) in a well‐supported clade (UFBoot = 98), distant from *S. veronicae* and *S. viticola* and resulting in polyphyly of the genus *Sorosphaerula*. A new combination of *S. radicalis* is therefore necessary.

## TAXONOMY

### *Hillenburgia radicalis* comb nov.

**Basionym:** *Sorosphaera radicale* Ivimey Cook & Schwartz.

**Synonyms:** *Sorosphaera radicalis* Cook & Schwartz; *Sorosphaerula radicalis* (Ivimey Cook & Schwartz) Neuh. & Kirchm.

Original description:

Soris sporarum globosis vel ellipsoideis cavis et membrana una amplexis. Soro sporarum vel amoeba solo iacente in pilo tumido radices. Diam. sporarum 3 μ; sororum 20–57 × 16–20 μ. Hab. In pilis radicis graminum diversorum ad Dunton Green, Kent, et Crawley, Sussex in Britannia.

### Morphology and life cycle of *Hillenburgia radicalis*

The life cycle of *H. radicalis* as described by Cook and Schwartz (1929) and Karling (1968) is expanded by findings of our study. Zoospores infect root hairs or epidermal cells at the root tip or the root elongation zone. Plasmodia can be found in epidermal cells or root hairs. Zoosporangia as well as sporosori are only observed in root hairs. This indicates that an infection might occur before epidermal cells develop into root hairs (Bibikova and Gilroy, 2002). Until now, it was anticipated that *H. radicalis* spends the whole life cycle in the root hairs of their host, however, we identified plasmodia and encysted spores near the root tip indicating that other cell types in the root can also be infected.

**FIGURE 3:**
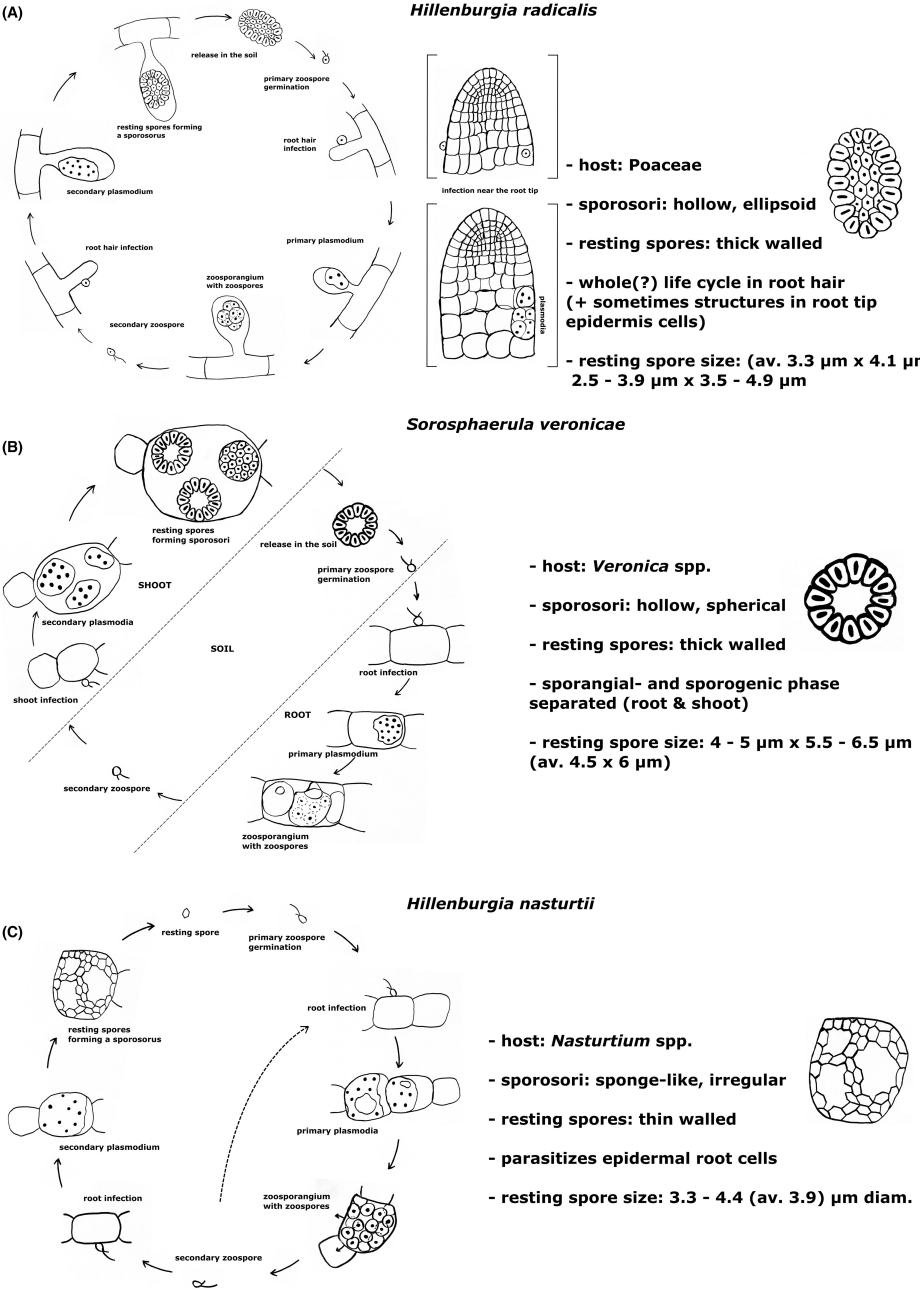
Comparison of *Hillenburgia radicalis*, *Sorosphaerula veronicae* and *Hillenburgia nasturtii*. The life cycle of *H. radicalis* (in accordance with literature [Cook and Schwartz 1929, Karling 1968] and observations made in this study); with additional observed divergences (in square brackets). *Hillenburgia radicalis* spends the whole life cycle in the root hairs of their host (various Poaceae). In this study, we found plasmodia and encysted spores in the epidermis near the root tip (shown in square brackets) (A). *Sorosphaerula veronicae* spends half of its life cycle in the root (sporangial phase), the other half in the shoot (sporogenic phase) of *Veronica* spp. In between those phases *S. veronicae* survives through thick walled resting spores in the soil, where it germinates into zoospores and re‐infects new host cells (Miller 1958, Karling 1968) (B). *Hillenburgia nasturtii* infects the epidermal root cells of *Nasturtium* spp. It leads to hypertrophy in infected cells and finally produce thin‐walled resting spores which form irregular, sponge‐like sporosori (Tomlinson 1958) (C). The main difference of the species is the form of the sporosorus: *H. radicalis* has hollow ellipsoid sporosori (A, right), *S. veronicae* has hollow spherical sporosori (B, right), and *H. nasturtii* has irregular, spongy sporosori (C, right)

A comparison of the life cycles of *H. radicalis, Sorosphaerula veronicae,* and *H. nasturtii* highlights differences and similarities (Figure 3). *S. veronicae* spends the sporangial phase of its life cycle in the root, while the sporogenic phase of the life cycle takes place in the shoot of *Veronica* spp. (Miller, 1958; Karling, 1968). The thick‐walled resting spores of *S. veronicae* are distinctively larger (4–5 μm × 5.5–6.5 μm, Kirchmair et al., 2005) than those of *H. radicalis* (4.1 μm × 3.3 μm). *Hillenburgia nasturtii* infects the epidermal root cells of *Nasturtium* spp. where it induces hypertrophy in infected cells. The sporosori are irregular and sponge‐like, the resting spores thin‐walled and have a size of 3.3–4.4 μm diameter (Tomlinson, 1958).

## DISCUSSION

We report the first collection of *Hillenburgia radicalis* in more than 50 years and the first record of the species in continental Europe. Previously there were only two reports of *H. radicalis*: the original description by Cook and Schwartz 1929 on samples from Sussex, the United Kingdom, and a collection from a university campus in California by Dr. J. T. Barret (Karling, 1968). We provide the first molecular barcode (18S rRNA, 1016 bp) of this species which will allow identification in metagenomic studies. No sequences with high similarity to *H. radicalis* could be found in the nucleotide collection (nr/nt) of Genbank. This lack of similar sequences suggests that this species is either (a) rare, (b) highly seasonal and/or ephemeral (similar to *Pseudoligniera verrucosa*, Hittorf et al., 2020), or (c) difficult to detect with commonly used PCR assays for metabarcoding studies (e.g., Neuhauser et al., 2014). The fact that *H. radicalis* has only been reported three times in nearly 100 years does not necessarily imply that this species is rare. On the contrary, it highlights the gap in our knowledge on phytomyxea, which do not form distinct hypertrophies and/or are not causing economical damage in food and feed crops. Targeted taxonomic studies and biodiversity inventories are therefore still paramount for a comprehensive understanding of phytomyxid biodiversity (Hittorf et al., 2020; Neuhauser et al., 2010; Neuhauser et al., 2014). Also, ephemeral and environmental factors influencing the presence and abundance of phytomyxea are not well understood in natural environments. Although we collected many additional Poa spp. Samples, the usually ubiquitous *Polymyxa graminis* could not be identified morphologically.

The historical taxonomic concepts are based on morphological traits such as the arrangement of the resting spores into sporosori and the host range (Braselton, 2001; Bulman and Braselton, 2014; Karling, 1968), but those differ from current molecular phylogenies (Hittorf et al., 2020; Neuhauser et al., 2014). This results in polyphyly of morphologically well‐defined genera. However, when looking into morphology in more detail similarities can become obvious—like in the case of the genus *Hillenburgia* the relatively similar size of the resting spores of *H. nasturtii* und *H. radicalis* and the clearly larger spore size of *S. veronicae*. Overall, 18S rRNA data and the morphological data provided here clearly support the reallocation of *H. radicalis*.
